# Youth Perceived Social Support and Symptom Distress: A Random-Intercept Cross-Lagged Panel Model

**DOI:** 10.1007/s10964-023-01859-7

**Published:** 2023-09-15

**Authors:** Eline M. Meuleman, William M. van der Veld, Odilia M. Laceulle, Paul T. van der Heijden, Maaike Verhagen, Elisa van Ee

**Affiliations:** 1 Behavioural Science Institute, Radboud University Nijmegen, Nijmegen, The Netherlands; 2 Department of Developmental Psychology, Utrecht University, Utrecht, The Netherlands; 3 Reinier van Arkel, Mental Health Institute, ‘s‐Hertogenbosch, The Netherlands

**Keywords:** Social support, Symptom distress, Youth, Reciprocal relations, Caregivers

## Abstract

Although social support and mental health associations have been extensively investigated, their reciprocal relations in vulnerable youth remain understudied. This study investigated the relations between perceived social support and symptom distress over time whilst differentiating between support from caregivers and significant others. The sample included 257 youth (79% self-identified women*, M*_age_ = 19.2, *SD* = 2.5) who were receiving mental health treatment. Using a Random-Intercept Cross-Lagged Panel Model, results revealed no significant concurrent associations, between-person effects, or cross-lagged effects. The autoregressive effects suggested that perceived social support from caregivers was relatively stable over time, while symptom distress and support from a significant other were not. In all, this study challenged the validity of the social causation and social erosion models in the context of perceived social support and symptom distress among vulnerable youth, revealing an absence of significant reciprocal associations. The stable nature of perceived social support from caregivers compared to support from significant others was highlighted. The study design, hypotheses, and target analyses were preregistered under https://osf.io/f4qpg.

## Introduction

The transition from childhood to (young) adulthood marks a time of essential changes. Biological, cognitive, social, and emotional transitions occur in all developmental domains. The neurological plasticity distinctive for youth makes them flexible to adapt to change, but also vulnerable to risky behavior and psychopathology (Rudolph et al., 2017). Indeed, most mental health problems—depression, substance disorders, anxiety disorders—evolve before the age of 25 (Solmi et al., 2022). The scientific evidence about the homotypic and heterotypic continuity of youth psychopathology into adulthood highlights the need to understand the mechanisms underlying youth mental health disorders (De Girolamo et al., 2012; Ranøyen et al., 2018). Therefore, this study focused on the interrelations between perceived social support and symptom distress among youth with mental disorders, shedding light on the complexities of youth psychopathology.

Several theoretical frameworks, such as the Contemporary Integrative Interpersonal Theory (Hopwood et al., 2023), form the basis for a wide consensus among researchers that interpersonal interactions shape the psychopathology of humans. Empirical research has shown that interpersonal relationships are crucial to help youth cope with stressful situations and to buffer psychological distress by providing them with social support (Camara et al., 2017). In the literature, social support is described as the actions undertaken by significant others, including family members, friends, or co-workers, to assist an individual who experiences distress (Thoits, 1986). However, individual differences might impact one’s perception of these social support functions (Stokes, 1985). Other research found that not the quality or quantity of received (actual) social support matters, but that perceived support, whether youth feel supported and understood by their close others, is most important (Eagle et al., 2019). Perceived social support facilitates self-compassion, well-being, and happiness and lowers perceived stress (Wilson et al., 2020). Overall, studies have indicated the significance of perceived social support for mental health, also among youth (Jakobsen et al., 2022).

Perceived social support and symptom distress are highly correlated in youth (e.g., Saikkonen et al., 2018). Symptom distress is an individual’s reaction to external and internal stressors, characterized by a mixture of psychological symptoms, such as anxiety and sadness (Ritsner et al., 2002). Two explanatory models hypothesize this relation between social support and symptom distress. Firstly, according to the social causation theory, perceived social support is an antecedent of mental well-being (Cohen & Wills, 1985). Those youth who perceive higher social support, are likely to have better mental well-being (Chu et al., 2010). This positive impact of social support on youth well-being can be attributed to the emotional, informational, and instrumental resources that it provides (Pearson, 1986). Secondly, the social erosion model states that mental health and distress are an antecedent of perceived social support (Kaniasty & Norris, 1993). That is, the perceived social support of persons with high levels of distress may decrease because of these symptoms. For example, individuals with depressive symptoms might have more complaints, inappropriate disclosure, and social inadequacy, which fosters erosion of their (received and perceived) support (Coyne, 1976), whereas youth with high levels of aggression might have more parent-child dyadic hostility and conflict-ridden peer relationships which erodes their perceived social support over time (Smokowski et al., 2016). In conclusion, previous research has indicated that perceived social support and symptom distress are closely linked and there are two main explanations for this association. The social causation theory posits that social support precedes mental well-being, while the social erosion model suggests that mental health disorders lead to a decline in social support.

Prior research into the social causation model explaining the relation between perceived social support and symptom distress in youth has found inconsistent results. The social causation model would predict that disruptions in perceived social support increase symptom distress in youth. Some empirical studies found that less perceived social support resulted in more depression symptoms (Barrera & Garrison-Jones, 1992; Stice et al., 2004; van Harmelen et al., 2016), somatic symptoms (Grigaitytė & Söderberg, 2021), anxiety symptoms (Calsyn et al., 2005) and aggressive behavior (Kumar et al., 2014). Receiving more social support in turn, positively affects mental well-being (Ringdal et al., 2020). However, this negative relation has not consistently been replicated within the literature. For example, a longitudinal study conducted by Ren et al. (2018) did not find that perceived social support affected depressive symptoms in adolescents. Thus, while the social causation model predicts that disruptions in perceived social support would increase symptom distress in youth, previous research has yielded inconsistent results.

Only a handful of studies investigated the social erosion model, which would predict that having symptom distress erodes perceived social support over time. In two longitudinal studies, having depressive symptoms significantly decreased adolescents’ (self-reported) peer support (Ren et al., 2018; Stice et al., 2004), whilst in another longitudinal study depressive symptoms in girls predicted decreases in family but not friend, social support (Slavin & Rainer, 1990). In addition, it was found that youth with higher levels of psychological distress reported lower levels of social support from family and friends (Banks & Weems, 2014) and that aggressive youth perceived less social support from their family (Wolff et al., 2014). However, two other studies showed no prospective relation between depressive symptoms and perceived social support (Joiner & Metalsky, 1995; Sheeber et al., 1997). In sum, empirical research into both the social causation and social erosion model explaining the relation between symptom distress and perceived social support in youth has shown conflicting results. In addition, researchers have urged for more longitudinal studies in outpatient samples that encompass a wider range of psychopathological measures beyond depression (Jakobsen et al., 2022; Rueger et al., 2016).

Furthermore, according to prior research, there should be increased attention on distinguishing between sources that provide social support (Gariepy et al., 2016; Pössel et al., 2018). The social network of youth is susceptible to change since there are many transitions during this phase: they build new friendships, acquire a larger network of peers, become more independent of their caretakers and form romantic relationships (Giordano et al., 2006). Therefore, it is important to consider differences between sources of support, since social support given by caregivers or (non-parental) significant others, such as friends, peers, or teachers, may have a differential impact on symptom distress. Some empirical research has indicated that non-parental others become increasingly important and influential providers of social support across development in young people (Buhrmester, 1996). A recent longitudinal study found that perceived social support from friends, but not parents, positively impacted adolescents’ well-being and negatively impacted depression and anxiety symptoms (Ringdal et al., 2020). However, other results demonstrated that adolescents with a lot of stress benefit the most from their family’s support (Pössel et al., 2018) and that a lack of parental support but not peer support led to a higher risk for developing major depression (Stice et al., 2004). Results on the second causal relationship, between perceived social support and symptom distress, have been more aligned and predict that depression promotes support erosion but only for peer support (Stice et al., 2004). This is consistent with theories suggesting that young people rely on caregivers to fulfill their fundamental needs and that caregivers are more likely to be enduring providers of social support for children and teenagers (Gariepy et al., 2016). In all, the reciprocal effects between perceived social support and symptom distress may differ based on the source of social support.

## Current Study

Research exploring the interrelations between perceived social support and psychopathologies has predominantly utilized cross-sectional methodologies, with a narrow focus on depression as the studied outcome. Furthermore, divergent findings have emerged concerning the potential differences between the social causation and social erosion models, contingent on the source of social support. In this study, these limitations were addressed by implementing a longitudinal investigation within a psychiatric outpatient sample, encompassing a broad psychopathological study outcome (symptom distress) and including youth perceived support of a caregiver and of a significant other. The corresponding research question was: Is there a reciprocal relation over time between symptom distress and (1) perceived social support of a caregiver and (2) perceived social support of a significant other in a sample of youth with mental disorders? It was hypothesized that there would be a reciprocal relation between perceived social support (from respectively a caregiver and a significant other) and symptom distress, which indicates that perceiving lower social support would result in higher symptom distress over time (social causation model) and having higher symptom distress would lead to lower perceived social support over time (social erosion model).

## Methods

### Participants

The sample consisted of 257 adolescents and young adults, including 203 self-identified women (79%) and 54 self-identified men (21%) between the ages of 12 and 23 years (*M*_age_ at T1 = 19.2, SD = 2.5). All youth had been referred to a mental health institution due to mental health disorders of varying degrees of severity and/or complexity and received a diagnosis and treatment in an outpatient treatment facility. Youth had diverse types of severe psychopathology, as classified by the DSM-5, with common mental health issues being mood and/or anxiety disorders often accompanied by comorbid features of personality disorders. Inclusion criteria were (1) having an age between 12 and 23 years old and (2) receiving outpatient treatment in a mental health institution. The exclusion criteria encompassed insufficient language proficiency, intellectual developmental disorders, and confusion due to a psychotic spectrum disorder. This study was approved by the ethical review committee of the Utrecht University Faculty for Social and Behavioral Sciences (FETC17-092).

### Procedure

Youth who were commencing treatment at an outpatient treatment facility were invited to participate by the APOLO (a Dutch language acronym for Adolescents and their Personality Development: A Longitudinal Study; Koster et al., 2022) team. Youth filled in online questionnaires every 6 months, resulting in a maximum data collection of six waves. The first assessment, at intake, was used for treatment indication as part of the standard diagnostic evaluation. During the other five assessments, which aimed to systematically evaluate the treatment, youth received the same measures (or a shortened test battery, see Koster et al. (2022)). Among these questionnaires were a few demographic questions (sex and age), the NRI-BSV and the SQ-48. At the beginning of the NRI-BSV questionnaire, youth were instructed to indicate which caregiver they would keep in mind while answering the questions about perceived social support. They were provided with the following options: biological, adoptive, foster, stepmother, stepfather, or other, and asked to select the caregiver of their own choice. Additionally, they were asked to choose a friend or significant other to keep in mind during the second part of the survey. The options for this were “my best friend (male)”, “my best friend (female)”, or “another important person”. Table 1 displays the caregivers and significant others who were specified by the youth as sources of support. Youth had 2 weeks (and 3 weeks at intake) before and after the intended assessment date to fill in the questionnaires. The research team made an effort in monitoring the follow-up assessments and notified youth to reduce drop-out.

**Table 1:** Descriptive information on caregiver and significant other

	*N*_T1_ (%)	*N*_T2_ (%)	*N*_T3_ (%)
Caregiver			
Mother	195 (75.9%)	149 (74.5%)	96 (78.7%)
Stepmother	1 (0.4%)	3 (1.5%)	0 (0%)
Father	48 (18.7%)	38 (19%)	24 (19.7%)
Stepfather	0 (0%)	0 (0%)	0 (0%)
Other	10 (3.9%)	10 (5%)	2 (1.6%)
Significant other			
Best friend (f)	106 (42%)	71 (35.5%)	24 (19.7%)
Best friend (m)	49 (19.2%)	44 (22%)	59 (48.4%)
Partner	50 (19.6%)	45 (22.5%)	13 (10.7%)
Family member	29 (11.4%)	19 (9.5%)	12 (9.8%)
Classmate	5 (2.0%)	0 (0%)	0 (0%)
Friend	4 (1.6%)	14 (7%)	3 (2.5%)
Co-worker	3 (1.2%)	1 (0.5%)	2 (1.6%)
Mentor	1 (0.4%)	2 (1%)	0 (0%)
Do not have	3 (1.2%)	4 (2%)	8 (6.6%)
Other	4 (1.6%)	0 (0%)	1 (0.8%)
Total	254 (100%)	200 (100%)	122 (100%)

### Measures

The Symptom Questionnaire-48 (SQ-48; Carlier et al., 2012) was used to measure symptom distress. The questionnaire consists of 48 items answered on a five-point scale (0 = “Never”, 4 = “Very often”). A mean score was determined by averaging the answers on all items of the subscales Depression (MOOD, six items), Anxiety (ANXI, six items), Somatization (SOMA, seven items), Agoraphobia (AGOR, four items), Aggression (AGGR, four items), Cognitive problems (COGN, five items) and Social Phobia (SOPH, five items). Answers on the remaining 37 items (all subscales but vitality and work which do not describe symptoms) were mean aggregated, with higher scores indicating greater symptom distress (Cronbach’s *α*_T1_ = 0.94; *α*_T2_ = 0.96; *α*_T3_ = 0.96).

Two items of the shortened version of the “Network of Relationships Inventory” (NRI-BSV; Furman and Buhrmester (2009); Dutch adaptation by van Aken & Hessels, 2012) were used to measure perceived social support. The total questionnaire consisted of eleven questions covering eight scales: seeks safe haven (one question), seeks secure base (one question), provides safe haven (one question), provides secure base (one question), companionship (one question), conflict (two questions), criticism (two questions) and antagonism (two questions). Perceived social support is in this research operationalized as whether a youth seeks a safe haven and/or secure base with a significant other. It is essential to distinguish that while providing social support to others or encountering negative interactions can undoubtedly influence social relationships, these specific interactions do not fall within the purview of perceived social support as defined in this study. The two items measuring seeking a safe haven and seeking a secure base were: “How much do you turn to this person for comfort and support when you are troubled about something?” and “How much does this person show support for your activities?” Each youth filled in the exact same set of questions twice: once about a caregiver and once about a non-parental significant other. Participants had the opportunity to report on different caregivers or significant others at various waves. The answers to these questions were indicated on a five-point Likert scale (1 = “Little or None”, 5 = “the Most”). The two items were mean aggregated, resulting in two scores for each youth: one score (based on the two items) reflecting a youth’s perceived social support of a caregiver and one score (based on the two items) indicating the perceived social support of a significant other. Higher scores indicated greater perceived support from a caregiver (Cronbach’s *α*_T1_ = 0.68; *α*_T2_ = 0.72; *α*_T3_ = 0.72) and a significant other (Cronbach’ *α*_T1_ = 0.65; *α*_T2_ = 0.65; *α*_T3_ = 0.71).

### Data Analyses

#### Attrition

The total longitudinal database of APOLO contained 688 participants in January 2023. At time point T4, T5, and T6, attrition was very high and the number of youth per wave was low (±30 participants), hence these waves were excluded from the analysis. Reasons for this drop-out were that treatment had already ended, treatment was completed at another institute or that youth were still in care but no longer willing to complete the questionnaires. These reasons were not known at the individual level. This study thus analyzed data from T1, T2, and T3. Perceived social support data was missing for respectively 12.4%, 67.6%, and 81.7% of the youth at T1, T2, and T3. Respectively 27.9%, 74.0%, and 84.3% of the youth at T1, T2, and T3 had missing data for the measure of symptom distress. The study included only youth who participated in the data collection for the NRI-BSV and/or SQ-48 questionnaires twice or thrice during the first three waves (T1: intake, T2: 6 months after intake, T3: 1 year after intake). Youth who only filled in one questionnaire at one wave were excluded from data analysis since for them no conclusions can be drawn regarding their changes over time. This resulted in a final dataset containing 257 youth. Youth who were excluded from the final analytic sample (*n* = 431) did not differ from those who were included (*n* = 257) with respect to symptom distress (*t*(494) = −0.16, *p* = 0.875), perceived social support of a caregiver at T1 (*t*(601) = −0.65, *p* = 0.517) or of a significant other (*t*(601) = −0.12, *p* = 0.906) at T1.

The guidelines of Nicholson et al. (2017) for attrition in developmental psychology were followed. *T-*tests were conducted to compare the mean values of the outcome variables for subjects with and without missing data in T2 and T3. Table 2 shows the attrition analyses. Youth who dropped out did not score significantly different on social support and symptom distress than those who did not. Therefore, it was assumed that the attrition is not systematic.

**Table 2:** Attrition analyses of the study variables at T2 and T3

	*M*(SD)- Stay_T2_	*n*	*M(*SD*)-* Out_T2_	*n*	*F*	*p*	*η*_*p*_^*2*^
Social support caregiver T1	3.2(1.1)	197	3.3(1.1)	57	0.71	0.40	0.00
Social support other T1	3.8(1.0)	197	3.6(1.0)	57	1.57	0.22	0.01
Symptom distress T1	1.9(0.7)	160	1.9(0.7)	59	0.41	0.84	0.00

	*M*(*SD*)- Stay_T3_	*n*	*M(SD)-* Out_T3_	*n*	*F*	*p*	*η*_*p*_^*2*^
Social support caregiver T1	3.4(1.1)	119	3.1(1.1)	135	4.4	0.04	0.02
Social support other T1	3.7(1.0)	119	3.7(1.0)	135	0.07	0.79	0.00
Symptom distress T1	1.9(0.8)	99	1.9(0.6)	120	0.00	0.99	0.00
Social support caregiver T2	3.4(1.2)	67	3.1(1.2)	133	4.97	0.03	0.02
Social support other T2	3.9(1.0)	67	3.8(1.1)	133	0.10	0.75	0.00
Symptom distress T2	1.7(0.8)	54	1.9(0.8)	111	1.57	0.21	0.01

The stay group represents the youth who participated in that wave, whereas the drop-out group did not fill in that wave. The first panel compares youth who filled in a questionnaire (social support caregiver, social support other, symptom distress) at T1 to the same set of youth who did or did not fill in that questionnaire at T2. The second panel compares youth responses at T1 or T2 with responses at T3.

#### Random-Intercept Cross-Lagged Panel Model

**Fig. 1:**
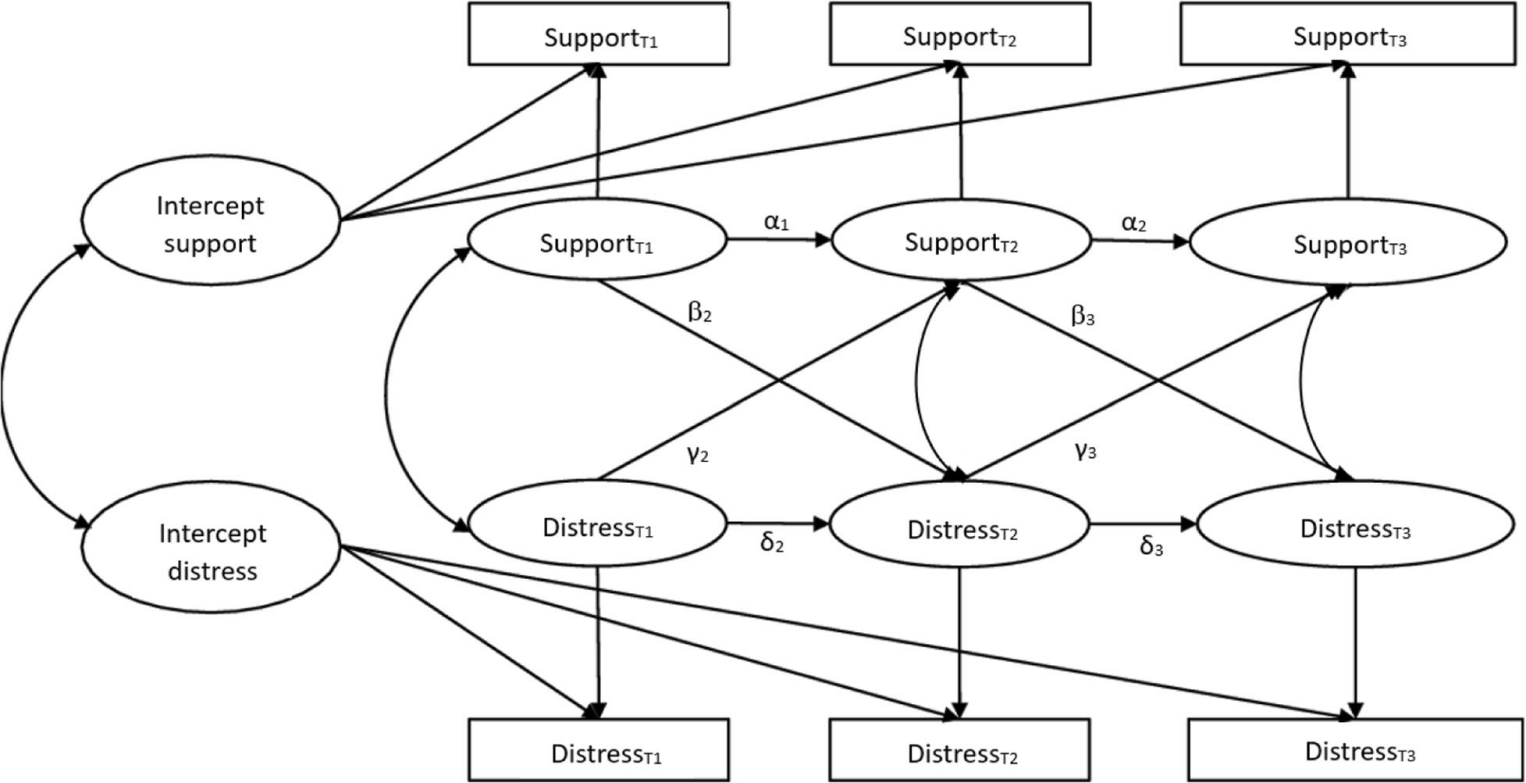
Conceptual RI-CLPM

Means, standard deviations, and inter-scale correlations between variables were computed in IBM SPSS 29 to assess the distribution of the data and the relations among the study variables. A random-intercept cross-lagged panel model (RI-CLPM) was used to examine the reciprocal relations between perceived social support and symptom distress (see Fig. 1 for the conceptual model). The RI-CLPM estimation using the Lavaan package (Rosseel, 2012) was performed in R (version 4.2.2; R Core Team, 2022).

RI-CLPM analyses are utilized to investigate dynamic effects in longitudinal data (Hamaker et al., 2015). In contrast to the traditional cross-lagged panel model, the RI-CLPM assumes that psychological constructs can change within an individual over time while also accounting for stable between-person differences. A RI-CLPM is thus able to disentangle effects on the within-person level (autoregressive paths, concurrent paths, cross-lagged paths) from effects on the between-person level (random intercepts). The autoregressive paths (α_2_ and α_3_ for perceived social support; δ_2_ and δ_3_ for symptom distress) capture the within-person changes of each construct over time. The concurrent paths represent the association between deviations in perceived support and symptom distress on the same measurement occasion (e.g., between Support_T1_ and Distress_T1_). The cross-lagged paths reflect the within-person reciprocal influence of these constructs over time. The paths β_2_ and β_3_ test whether social support predicts symptom distress over time (social causation theory), whereas γ_1_ and γ_2_ are in line with the social erosion theory, with symptom distress predicting social support over time. The random intercepts are stable, time-invariant between-person differences in social support and symptom distress. Factor loadings for these random intercepts were fixed to one. In addition, two covariates were included in the analyses, sex and age. The effects of these covariates on the observed outcome variables were estimated at each wave. In sum, rather than treating psychological constructs as overall measures that vary around their temporary group means, the RI-CLPM separates the variance of the between-person differences of the constructs across time (Hamaker et al., 2015).

Preliminary analyses found no problematic deviations from normality (by inspection of histograms, skewness, and kurtosis) for symptom distress. However, values of support from a caregiver and support from a significant other were negatively skewed and most of them had a negative kurtosis, with only support from another at T2 deviating with a positive kurtosis. A robust estimator method (‘MLR’) was employed to correct for deviations from normality, while full information maximum likelihood estimation was used to handle missing data.

To evaluate the model fit, the Chi-square (χ2), comparative fit index (CFI), root mean square error of approximation (RMSEA) and standardized root mean square residual (SRMR) were used as goodness-of-fit indices (Hu & Bentler, 1999). Models that have CFI > 0.90, RMSEA < 0.08, and SRMR < 0.08 were considered to have an acceptable fit, while models with CFI > 0.95, RMSEA < 0.05, and SRMR < 0.05 were evaluated to have a good fit. For the Chi-square test, the significance test should show *p* ≥ 0.05 (because the null hypothesis is that the model fits the data; this should not be rejected).

## Results

### Descriptive Analyses

The means and standard deviations of the variables in this study are shown in Table 3. In addition, a series of within-subject *t*-tests were conducted to investigate whether there were significant differences between two time points (i.e., T1 and T2 or T2 and T3). There were no significant differences in symptom distress between T1 and T2, *t*(159) = 1.78, *p* = 0.077, or between T2 and T3, *t*(53) = 1.16, *p* = 0.253. Similarly, no significant differences were observed between T1 and T2, *t*(196) = −0.12, *p* = 0.903 and T2 and T3, *t*(66) = 0.83, *p* = 0.409 for perceived social support from a caregiver. Lastly, results indicated that perceived social support of a significant other did not differ between T1 and T2, *t*(196) = −1.37, *p* = 0.172, nor between T2 and T3, *t*(66) = 1.01, *p* = 0.319.

**Table 3:** Univariate statistics of the study variables at T1 and T2

	Mean	SD	*N*	Missing
Symptom distress T1	1.9	0.7	219	38
Symptom distress T2	1.8	0.8	165	92
Symptom distress T3	1.8	0.8	104	153
Support from caregiver T1	3.2	1.1	254	3
Support from caregiver T2	3.2	1.2	200	57
Support from caregiver T3	3.4	1.1	122	135
Support from significant other T1	3.7	1.0	254	3
Support from significant other T2	3.9	1.0	200	57
Support from significant other T3	3.8	1.0	122	135

Table 4 presents the Pearson product correlations between the focal variables. The findings revealed that most of the time, symptom distress had a significant negative association with perceived social support from a caregiver. When examining the associations between symptom distress and perceived social support from a significant other, results showed that most correlations were weak and non-significant. Perceived social support of a caregiver and perceived social support of a significant other were positively associated, and in most cases this correlation was significant.

**Table 4:** Pearson product correlations

	1	2	3	4	5	6	7	8	9	10
1. Distress_T1_										
2. Distress_T2_	0.62^**^									
3. Distress_T3_	0.66^**^	0.78^**^								
4. Support-C_T1_	−0.13	−0.19^*^	−0.28^**^							
5. Support-C_T2_	−0.22^**^	−0.26^**^	−0.46^**^	0.72^*^						
6. Support-C_T3_	−0.09	−0.29^*^	−0.22^*^	0.60^**^	0.72^**^					
7. Support-O_T1_	−0.00	−0.10	−0.02	0.17^**^	0.15^*^	0.14				
8. Support-O_T2_	0.01	−0.06	−0.09	0.17^**^	0.25^**^	0.08	0.45^**^			
9. Support-O_T3_	0.05	−0.14	−0.11	0.27^**^	0.12	0.33^**^	0.45^**^	0.58^**^		
10. Age	0.02	−0.09	−0.15	−0.15^*^	−0.19^**^	−0.14	0.10	0.05	0.20*	
11. Gender^a^	0.18^**^	0.05	0.06	0.08	0.13	0.14	0.11	0.18^*^	0.24^**^	0.02

*p < 0.05. **p < 0.01

^a^0 = self-identified male and 1 = self-identified female. Support-C stands for perceived social support of a caregiver; Support-O stands for perceived social support of a significant other. Pairwise deletion was used with samples ranging from *n* = 54 to *n* = 254.

### Random-Intercept Cross-Lagged Panel Model

RI-CLPM analyses were conducted to examine how perceived social support and symptom distress influence each other over time while taking into account individual differences in starting points and changes in those variables. The model was estimated twice, once with symptom distress and perceived social support by the caregiver and once with symptom distress and perceived social support of a significant other.

**Fig. 2:**
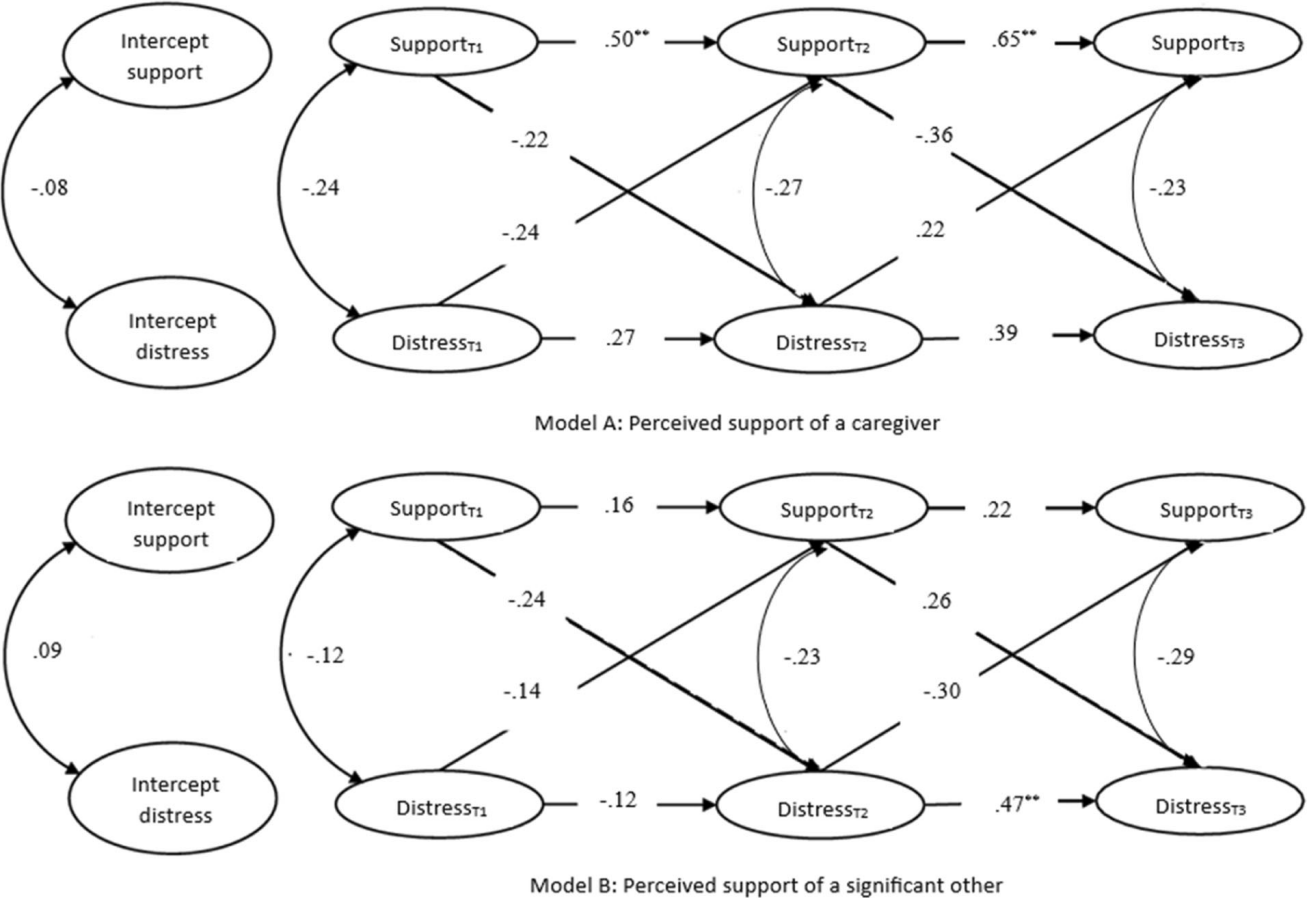
Results of the RI-CLPM. *Note*: Within and between-person effects for Model A (symptom distress and perceived support of a caregiver) and Model B (symptom distress and perceived support of a significant other). The estimates were standardized. ^*^p < 0.05. ^**^p < 0.01

The model with perceived support of a caregiver (Fig. 2, Model A) fitted the data well; robust *χ*^2^(1) = 1.02, *p* = 0.313, robust CFI = 1.00, robust RMSEA = 0.01, robust SRMR = 0.01. At the within-person level, the autoregressive effects of support were significant (α_1_ and α_2_). This indicates that occasions on which a youth scored above the expected score in perceived social support of a caregiver were likely to be followed by occasions on which the scores were above the expected score again in the following time points. The autoregressive effects of symptom distress were not significant (δ_3_ and δ_4_), meaning that within-person deviations in symptom distress were not enduring over time. Concurrent associations between support and distress were also not significant at all waves. Within-person changes (deviations from a youth’s expected score) in support and within-person changes in symptom distress were not associated cross-sectionally. No cross-lagged effects were found; the estimates of β_2_, β_3_, γ_2_ and γ_3_ were all non-significant, implying an absence of (statistically significant) reciprocal relations. At the between-person level, no significant correlation between the random intercepts of social support and symptom distress was found, indicating that—at the group level—youth who had higher levels of perceived social support of a caregiver did not report less symptom distress.

The model with perceived support of a significant other (Fig. 2, Model B) fitted the data well; robust *χ*^2^(1) = 1.52, *p* = 0.217, robust CFI = 1.00, robust RMSEA = 0.04, robust SRMR = 0.01. One significant effect was found: an autoregressive effect of distress from T2 to T3 (δ_3_). This indicates that only at T2, symptom distress levels above a youth’s mean predicted higher levels of distress at T3. The other autoregressive effects, concurrent associations, cross-lagged effects, and between-person associations were not significant.

These models, though not depicted in Fig. 2, controlled for age and gender. Looking at the role of these covariates in Model A, results showed that age significantly predicted the perceived social support of a caregiver at T1 (Standardized Estimate = −0.19, *p* = 0.011). This indicates that younger youth perceived their caregiver as more supportive at the first measurement (only). Gender significantly influenced symptom distress at T1 (Standardized Estimate = 0.28, *p* = 0.005), indicating that on average self-identified women had a higher level of symptom distress than self-identified men at the first measurement. Other results for age and gender were non-significant.

For Model B, age significantly predicted symptom distress at T3 (Standardized Estimate = −0.26, *p* = 0.036), showing that younger youth generally had higher levels of symptom distress at the third measurement. Gender significantly influenced symptom distress at T1 (Standardized Estimate = 0.34, *p* = 0.009) and perceived social support of a significant other at T2 (Standardized Estimate = 0.25, *p* = 0.027) and T3 (Standardized Estimate = 0.33, *p* = 0.016). Self-identified women had higher levels of symptom distress at T1 and perceived their significant other as more supportive at T2 and T3.

Two exploratory analyses were performed. The first investigated the two models (Model A & Model B) for each of the symptom distress subscales separately. The pattern of results mostly aligned with the results of the models presented here. No significant consistent cross-lagged effects were found over time between perceived social support and the parts of symptom distress. A second exploratory analysis investigated whether the pattern of results found differed for adolescents and emerging adults, as youth tend to shift their reliance for support on parents towards friends and romantic partners over time. In the present study, 63 adolescents (aged 12–17) and 194 emerging adults (aged 18–23) participated. Models were run again for emerging adults only, since for adolescents models did not converge due to power insufficiencies. The pattern of results for emerging adults was identical to the total sample in both Model A and Model B, with standardized estimates being similar.

## Discussion

Interpersonal relationships are crucial to understand youth development and psychopathology (Camara et al., 2017). According to the social causation theory and the social erosion theory, perceived social support and symptom distress are highly associated (Cohen & Wills, 1985; Kaniasty & Norris, 1993). Empirical studies focusing on the social causation and social erosion theories (e.g., Ren et al., 2018; Ringdal et al., 2020), found mixed support for these theories. The results of the present study shed new light on the relations between perceived social support and symptom distress over time in a sample of 257 vulnerable youth, whilst controlling for gender and age.

This study represents a novel approach to the literature by investigating the reciprocal relations between perceived social support and symptom distress in youth. Prior investigations predominantly utilized cross-sectional designs, limiting the ability to draw conclusions about the direction of effects (Gariepy et al., 2016; Rueger et al., 2016). By using a RI-CLPM approach, this study examined the social causation and social erosion theories while disentangling within-person from between-person effects. The findings indicated that there was no evidence supporting the concurrent associations, cross-lagged paths, or between-person associations. The absence of concurrent associations and between-person associations is further accentuated by the weak correlations between both variables in the present study. There were some medium correlations between perceived social support of a caregiver and symptom distress, but all other correlations were weak. Moreover, no cross-lagged relations between perceived social support (of a caregiver and a significant other) and symptom distress were found. These findings contrast with several prior studies that did find support for the social causation and social erosion theories (e.g., Ringdal et al., 2020) and align with some other studies that also did not find significant relations (e.g., Ren et al., 2018). The exploratory analyses demonstrated that the results remained consistent for emerging adults aged 18–23, and additionally, no consistent cross-lagged effects over time were observed between perceived social support and any of the subscales. In all, this study uniquely investigated the temporal and directional effects between perceived social support and symptom distress in youth, utilizing a RI-CLPM approach to disentangle within- and between-person effects, revealing a lack of evidence for concurrent associations, cross-lagged paths, and between-person associations.

The absence of significant findings may be due to both methodological and theoretical factors. First of all, from a methodological point of view, it is notable that even though the standardized estimates in the main model were quite large (e.g., cross-lagged effects of −0.36 and 0.22; Fig. 2), the effects remained non-significant. The samples used to estimate the RI-CLP models varied in size, ranging from *N* = 254 for perceived social support at T1 to *N* = 104 for symptom distress at T3 (Table 3). Due to the lower sample size, particularly in wave three, it is possible that the statistical power was insufficient to detect significant results in the RI-CLPM. To evaluate these possible power insufficiencies, further exploratory analyses were conducted using simplified models, including a cross-lagged panel model without random intercepts and a cross-lagged panel model with two waves. Although these simplified models likely presented higher statistical power, they also did not demonstrate any significant cross-lagged effects. Future research should, however, replicate these findings with larger samples and more follow-up measures whilst utilizing a statistical method that can shed light on the direction of effects.

Besides, it might be possible that perceived social support and symptom distress (as well as other factors like aggression, agoraphobia, anxiety, cognitive problems, mood, somatic complaints, and social phobia) do not exhibit reciprocal influences on each other among youth in treatment. Firstly, in the present study, it was observed that the means of symptom distress remained relatively constant over time. The absence of a stable decline in symptom distress, which is further accentuated by the lack of significant stability paths of symptom distress and its subscales, could explain the null findings since perceived social support can not account for (at least a portion of) any change in symptom distress. Secondly, the absence of associations, correlations, and reciprocal relations might be explained by the study’s sample. The current sample involved youth seeking and/or starting mental health therapy due to (severe) mental health disorders. Based on the social erosion theory, it could be that these youth’s perceived social support had already been eroded before they started treatment as they might have endured prolonged periods of high distress prior to starting treatment. Starting treatment might enhance perceived social support over time because positive experiences in therapy (e.g., building a therapeutic relationship, developing trust, disclosing thoughts and feelings) can serve as a model for youth to generalize to their relationships outside of therapy (Follette et al., 1996; Thompson & Goodvin, 2016). Prior research has, however, shown that the (newly built-up) effects of perceived support require some time to manifest (i.e., a sleeper effect of support; Torsheim et al., 2003). Following youth who are in treatment for 1 year in total might, therefore, be too short to capture all reciprocal effects, especially because there are often waiting times between intake and the start of treatment. In summary, the non-significant findings may be explained by two theoretical factors: the heterogeneous nature of symptom distress as an outcome and the sleeper effect of support.

This study’s results on the autoregressive effects indicated that perceived social support from a caregiver was stable over time in all models (for the whole of symptom distress and its subscales), whereas support from a significant other was not. Young individuals who perceived high levels of support from their caregiver at one time point were likely to perceive similar levels of support at a later point, which supports the theory that caregivers are a dependable and stable source of social support for children and adolescents despite changing relationships (Gariepy et al., 2016). Conversely, the youth’s perceived support of a significant other was fluctuating. According to prior research, this instability could be due to changes in peer networks or the possibility of oscillating between feelings of acceptance and rejection (Paus et al., 2008; Rudolph et al., 2017; Stice et al., 2004). In addition, some youth who are in treatment no longer participate in social and community life (such as school) and, as a result, have (temporarily) fewer social contacts. These differences in stability between sources of support can also be observed through percentages obtained from the questionnaire responses. In particular, out of 64 youth who completed all questionnaires in all three waves of the study, 72% consistently reported the perceived support of the same caregiver for each of the three waves, whereas 60% of the youth consistently reported about the same significant other. It is recommended that future research investigates potential variations in perceived social support, such as examining whether the quality or stability of a relationship has a more significant impact on perceived social support than the source of support.

The autoregressive effects of symptom distress were inconsistent. In the main model and some subscales, there were significant autoregressive paths, mostly from T2 to T3 in Model B. Thus, in general, symptom distress fluctuated between T1 and T2, and remained more stable between T2 and T3 (with *p* = 0.08 in the Model A of the main analysis and *p* < 0.01 in the Model B). One possible explanation that could account for these findings is the waiting time between intake and the start of treatment. The initial measurement took place at intake, which is often followed by a waiting period before the actual start of treatment. Therefore, at T2, some youth were just beginning their treatment, and this could clarify why treatment benefits, such as stable reductions in symptom distress, were only noticeable between T2 and T3.

The finding that caregivers were a relatively stable form of support for youth who are in treatment, whilst perceived support from significant others such are peers and siblings was more fluctuating, holds practical implications. Previous research has pointed out the lack of support for parent/family involvement in therapy for young individuals (Rueger et al., 2016), despite attachment theorists and researchers demonstrating the importance of family support for children of all ages (Bowlby, 1969). Hence, caregivers could be educated about their role as a significant source of support and could be provided with resources to support their children in developmentally appropriate ways. Such interventions should aim to reduce negative behaviors like hostility and rejection and provide encouragement and support from caregivers to their children (McLeod et al., 2007; Rueger et al., 2016). Possible avenues for future research include examining the interrelations between conflict, perceived social support and mental health-related outcomes, distinguishing between perceived social support from a father or mother and investigating whether distinct groups of youth are characterized by consistently low or high levels of perceived social support. Furthermore, the timing of assessments has an important role in longitudinal designs, and future studies need to identify the optimal time points to capture changes in perceived social support and symptom distress among youth undergoing treatment.

Limitations of this study should be considered. A first possible limitation is the measure of perceived social support, which relied on only two items. The questionnaire showed acceptable reliability estimates but could have been improved by including more items for more reliable results. Secondly, the present study used two follow-up assessments at 6-month intervals, which may have limited the ability to capture acute dynamic changes occurring within shorter timeframes. Therefore, future research should consider utilizing more frequent assessments, such as weekly or monthly intervals. Thirdly, in the context of the current study, which includes youth aged between 12 and 24 years, the within-person dynamics of perceived social support may have changed as a function of age. However, the RI-CLPMs did control for age and a sensitivity analysis with emerging adults showed no distinctions from the outcomes observed in the total sample. Future research endeavors should consider clustering participants into distinct age groups to elucidate the within-person fluctuations that may arise within these groups.

## Conclusion

Although research underscores the role of social support in youth’s mental well-being, there is limited research testing the complex interplay between perceived support from various sources and symptom distress over time. This study employed a rigorous and relatively new analysis to examine the relations between perceived social support from caregivers and significant others and symptom distress, contributing to the understanding of the social causation and social erosion models. By disentangling within-person and between-person effects, this study provided novel insights into the directionality of these effects. This study’s findings challenged the validity of the social causation and social erosion models in the context of perceived social support and symptom distress in vulnerable youth, as no significant reciprocal associations were observed. The presence of an autoregressive effect did highlight the relative stability of perceived social support from caregivers compared to support from significant others. In conclusion, this study found no reciprocal relations between perceived social support and symptom distress, and future research is needed to delve deeper into the causes and consequences of mental well-being among youth.

## Data Availability

This manuscript’s data will not be deposited.
